# (*E*)-4-[(4-Nitro­phen­yl)diazen­yl]phenyl anthracene-9-carboxyl­ate

**DOI:** 10.1107/S1600536808034958

**Published:** 2008-11-08

**Authors:** Mark A. Rodriguez, Jessica L. Nichol, Thomas Zifer, Andrew L. Vance, Bryan M. Wong, François Léonard

**Affiliations:** a PO Box 5800, MS 1411, Sandia National Laboratories, Albuquerque, New Mexico 87185, USA; b Department of Chemistry, Indiana University of Pennsylvania, Indiana, Pennsylvania 15705, USA; c PO Box 969, MS 9403, Sandia National Laboratories, Livermore, California 94551, USA; d PO Box 969, MS 9161, Sandia National Laboratories, Livermore, California 94551, USA

## Abstract

In the title compound, C_27_H_17_N_3_O_4_, the azo group displays a *trans* conformation and the dihedral angles between the central benzene ring and the pendant anthracene and nitro­benzene rings are 82.94 (7) and 7.30 (9)°, respectively. In the crystal structure, weak C—H⋯O hydrogen bonds, likely associated with a dipole moment present on the mol­ecule, help to consolidate the packing.

## Related literature

This structure is similar to the perviously reported compound (*E*)-2-{Eth­yl[4-(4-nitro­phenyl­diazen­yl)phen­yl]amino}ethyl anthracene-9-carboxyl­ate (Rodriguez, *et al.*, 2008 ▶). For general background, see: Atassi *et al.* (1998 ▶); Becke (1993 ▶).
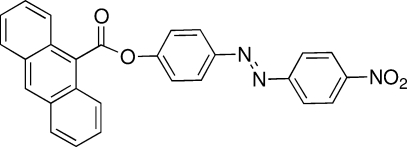


## Experimental

### 

#### Crystal data

C_27_H_17_N_3_O_4_
*M* *_r_* = 447.44
Monoclinic, 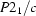

*a* = 13.525 (2) Å
*b* = 8.6011 (14) Å
*c* = 18.956 (3) Å
β = 109.322 (3)°
*V* = 2080.9 (6) Å^3^
*Z* = 4
Mo *K*α radiation
μ = 0.10 mm^−1^
*T* = 173 (2) K
0.20 × 0.18 × 0.05 mm

#### Data collection

Bruker SMART CCD area-detector diffractometer
Absorption correction: multi-scan (*SADABS*; Sheldrick, 1999 ▶) *T*_min_ = 0.980, *T*_max_ = 0.995
14511 measured reflections
3665 independent reflections
2752 reflections with *I* > 2σ(*I*)
*R*_int_ = 0.038

#### Refinement

*R*[*F* ^2^ > 2σ(*F* ^2^)] = 0.043
*wR*(*F* ^2^) = 0.100
*S* = 1.03
3665 reflections
307 parameters
H-atom parameters constrained
Δρ_max_ = 0.39 e Å^−3^
Δρ_min_ = −0.16 e Å^−3^

### 

Data collection: *SMART* (Bruker, 2001 ▶); cell refinement: *SMART* (Bruker, 2001 ▶); data reduction: *SAINT-Plus*; program(s) used to solve structure: *SHELXTL* (Sheldrick, 2008 ▶); program(s) used to refine structure: *SHELXTL*; molecular graphics: *XSHELL* (Bruker, 2001 ▶); software used to prepare material for publication: *SHELXTL*.

## Supplementary Material

Crystal structure: contains datablocks I, global. DOI: 10.1107/S1600536808034958/hb2827sup1.cif

Structure factors: contains datablocks I. DOI: 10.1107/S1600536808034958/hb2827Isup2.hkl

Additional supplementary materials:  crystallographic information; 3D view; checkCIF report

## Figures and Tables

**Table 1:** Hydrogen-bond geometry (Å, °)

*D*—H⋯*A*	*D*—H	H⋯*A*	*D*⋯*A*	*D*—H⋯*A*
C26—H26⋯O2^i^	0.95	2.54	3.273 (3)	134
C17—H17⋯O4^ii^	0.95	2.57	3.509 (3)	169

Symmetry codes: (i) 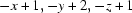; (ii) 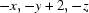.

**Fig. 1.**
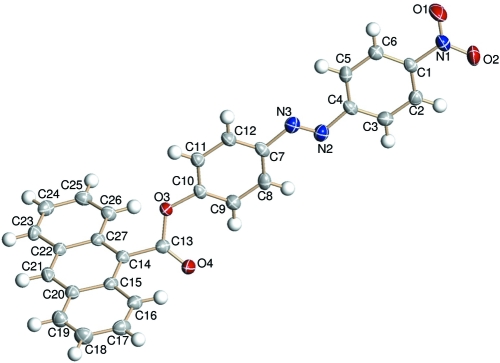
The molecular structure of (I) with 50% probability displacement ellipsoids for non-H atoms.

**Fig. 2.**
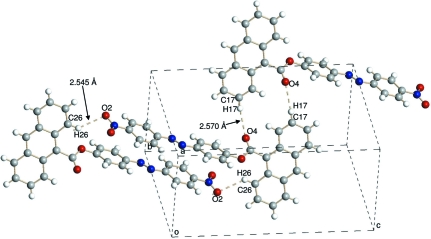
A packing diagram of (I) illustrating weak C—H···O hydrogen-bond interactions associated with terminal oxygen atoms O2 and O4.

## supplementary crystallographic information

### Comment

Atassi *et al.* (1998) has documented photoisomerization of the azobenzene in Disperse Red 1 (DR1) to a *cis* conformation under UV light, with decay back to the equilibrium *trans* species with removal of the UV light. In this manuscript we present another compound, (I), containing a *trans* azobenzene conformational state (Fig. 1). The displacement ellipsoids for most of the atoms are well defined. However, the O1 and O2 atoms at the termination of the nitroazobenzene unit do show subtle enlargement.

Figure 2 shows a packing arrangement and intermolecular interactions for (I). The nitroazobenzene portion is nearly planar as is the anthracene portion of the molecule. The anthracene is rotated from the nitroazobenzene through the carboxyl group. The title compound displays a head-to-toe configuration *via* weak C—H···O bonds as shown in Figure 2.  Specifically, an O2 atom of one molecule makes a weak bond to H26 of the neighboring molecule with a bond length of 2.55 Å. The calculated dipole moment for a molecule of (I) is 7.6806 Debye using the B3LYP functional (Becke, 1993) with the 6–311 G(d,p) triple-zeta basis. This dipole moment likely drives the head-to-toe alignment of the molecules as illustrated in Figure 2.

The structure of (I) is similar in form to that of the previously reported ester (*E*)-2-{ethyl[4-(4-nitrophenyldiazenyl)phenyl]amino}ethyl anthracene-9-carboxylate (Rodriguez, *et al.*, 2008), with the subtle difference relating to the absence of the ethyl-amino ligand in (I). As with the aformentioned compound, intermolecular interactions for the title compound are exclusively C—H···O in nature (Table 2). An additional interaction which bridges molecules in the *a* axis direction is also shown in Figure 2. This weak hydrogen bond is between the terminal carboxyl oxygen O4 and the neighboring H17 atom. The hydrogen bond shows a length of 2.57 Å and symmetrically bonds the two H atoms of the anthracene of each molecule.

### Experimental

The title compound was synthesized from 9-anthracenecarboxylic acid and 4-(4-nitrophenyl)azophenol *via* a dicyclohexylcarbodiimide esterification in anhydrous dichloromethane. After filtration of insoluble side products and removal of solvent by rotary evaporation, the crude product was dissolved in dichloromethane and filtered through a silica gel plug. Evaporation of the solvent gave a red powder that was characterized by 1*H*-NMR, UV/Vis and FTIR. Red crystals of (I) were obtained by recrystallization from hot dichloromethane.

### Crystal data

**Table:**

C_27_H_17_N_3_O_4_	*F*_000_ = 928
*M**_r_* = 447.44	*D*_x_ = 1.428 Mg m^−^^3^
Monoclinic, *P*2_1_/*c*	Mo *K*α radiation λ = 0.71073 Å
Hall symbol: -P 2ybc	Cell parameters from 100 reflections
*a* = 13.525 (2) Å	θ = 1.6–25.0º
*b* = 8.6011 (14) Å	µ = 0.10 mm^−^^1^
*c* = 18.956 (3) Å	*T* = 173 (2) K
β = 109.322 (3)º	Plate, red
*V* = 2080.9 (6) Å^3^	0.20 × 0.18 × 0.05 mm
*Z* = 4	

### Data collection

**Table:**

Bruker SMART CCD area-detector diffractometer	3665 independent reflections
Radiation source: fine-focus sealed tube	2752 reflections with *I* > 2σ(*I*)
Monochromator: graphite	*R*_int_ = 0.038
Detector resolution: 0 pixels mm^-1^	θ_max_ = 25.0º
*T* = 173(2) K	θ_min_ = 1.6º
φ and ω scans	*h* = −16→16
Absorption correction: multi-scan(SADABS; Sheldrick, 1999)	*k* = −10→10
*T*_min_ = 0.980, *T*_max_ = 0.995	*l* = −21→22
14511 measured reflections	

### Refinement

**Table:**

Refinement on *F*^2^	Secondary atom site location: difference Fourier map
Least-squares matrix: full	Hydrogen site location: inferred from neighbouring sites
*R*[*F*^2^ > 2σ(*F*^2^)] = 0.043	H-atom parameters constrained
*wR*(*F*^2^) = 0.100	*w* = 1/[σ^2^(*F*_o_^2^) + (0.0388*P*)^2^ + 0.7361*P*] where *P* = (*F*_o_^2^ + 2*F*_c_^2^)/3
*S* = 1.03	(Δ/σ)_max_ = 0.001
3665 reflections	Δρ_max_ = 0.39 e Å^−^^3^
307 parameters	Δρ_min_ = −0.16 e Å^−^^3^
Primary atom site location: structure-invariant direct methods	Extinction correction: none

### Special details

**Table:**

Geometry. All e.s.d.'s, except the e.s.d. in the dihedral angle between two least-square (l.s.) planes, are estimated using the full covariance matrix. The cell e.s.d.'s are taken into account individually in the estimation of e.s.d.'s in distances, angles and torsion angles; correlations between e.s.d.'s in cell parameters are only used when they are defined by crystal symmetry. An approximate (isotropic) treatment of cell e.s.d.'s is used for estimating e.s.d.'s involving l.s. planes.
Refinement. Refinement of *F*^2^ against ALL reflections. The weighted *R*-factor *wR* and goodness of fit *S* are based on *F*^2^, conventional *R*-factors *R* are based on *F*, with *F* set to zero for negative *F*^2^. The threshold expression of *F*^2^ > σ(*F*^2^) is used only for calculating *R*-factors(gt) *etc*. and is not relevant to the choice of reflections for refinement. *R*-factors based on *F*^2^ are statistically about twice as large as those based on *F*, and *R*- factors based on ALL data will be even larger.

### Fractional atomic coordinates and isotropic or equivalent isotropic displacement parameters (Å^2^)

**Table:**

	*x*	*y*	*z*	*U*_iso_*/*U*_eq_	
N1	0.69229 (14)	1.52655 (19)	0.65761 (9)	0.0352 (4)	
N2	0.44775 (13)	1.12430 (19)	0.43308 (9)	0.0350 (4)	
N3	0.48826 (13)	1.08369 (18)	0.38663 (9)	0.0333 (4)	
O1	0.78660 (13)	1.5368 (2)	0.66951 (9)	0.0581 (5)	
O2	0.64573 (13)	1.60250 (18)	0.69126 (8)	0.0486 (4)	
O3	0.26778 (10)	0.69438 (15)	0.15211 (7)	0.0304 (3)	
O4	0.10043 (11)	0.77469 (17)	0.12517 (8)	0.0416 (4)	
C1	0.63159 (15)	1.4180 (2)	0.59981 (10)	0.0272 (4)	
C2	0.52768 (15)	1.3952 (2)	0.59086 (11)	0.0322 (5)	
H2	0.4957	1.4476	0.6218	0.039*	
C3	0.47087 (16)	1.2945 (2)	0.53590 (11)	0.0334 (5)	
H3	0.3993	1.2749	0.5295	0.040*	
C4	0.51755 (15)	1.2218 (2)	0.49006 (10)	0.0289 (5)	
C5	0.62352 (16)	1.2433 (2)	0.50109 (11)	0.0307 (5)	
H5	0.6556	1.1907	0.4703	0.037*	
C6	0.68173 (16)	1.3411 (2)	0.55702 (11)	0.0304 (5)	
H6	0.7545	1.3555	0.5660	0.037*	
C7	0.42162 (15)	0.9862 (2)	0.32817 (10)	0.0298 (5)	
C8	0.31857 (16)	0.9475 (2)	0.31890 (11)	0.0326 (5)	
H8	0.2860	0.9874	0.3524	0.039*	
C9	0.26266 (16)	0.8502 (2)	0.26061 (11)	0.0316 (5)	
H9	0.1924	0.8218	0.2543	0.038*	
C10	0.31210 (15)	0.7960 (2)	0.21211 (10)	0.0273 (4)	
C11	0.41510 (15)	0.8342 (2)	0.22141 (11)	0.0296 (5)	
H11	0.4480	0.7949	0.1879	0.036*	
C12	0.46943 (16)	0.9293 (2)	0.27953 (11)	0.0318 (5)	
H12	0.5401	0.9560	0.2862	0.038*	
C13	0.16358 (15)	0.6938 (2)	0.11132 (11)	0.0281 (4)	
C14	0.14418 (14)	0.5854 (2)	0.04656 (10)	0.0258 (4)	
C15	0.09289 (14)	0.6443 (2)	−0.02570 (11)	0.0265 (4)	
C16	0.05487 (14)	0.8006 (2)	−0.04087 (12)	0.0313 (5)	
H16	0.0626	0.8699	−0.0004	0.038*	
C17	0.00814 (16)	0.8515 (2)	−0.11171 (12)	0.0383 (5)	
H17	−0.0159	0.9559	−0.1201	0.046*	
C18	−0.00531 (17)	0.7513 (3)	−0.17329 (12)	0.0426 (6)	
H18	−0.0391	0.7882	−0.2227	0.051*	
C19	0.02967 (16)	0.6035 (2)	−0.16205 (12)	0.0381 (5)	
H19	0.0206	0.5375	−0.2039	0.046*	
C20	0.07981 (14)	0.5446 (2)	−0.08895 (11)	0.0287 (5)	
C21	0.11527 (14)	0.3921 (2)	−0.07705 (11)	0.0296 (5)	
H21	0.1070	0.3269	−0.1191	0.036*	
C22	0.16242 (14)	0.3316 (2)	−0.00585 (11)	0.0256 (4)	
C23	0.19358 (15)	0.1725 (2)	0.00493 (12)	0.0309 (5)	
H23	0.1821	0.1069	−0.0373	0.037*	
C24	0.23912 (15)	0.1134 (2)	0.07422 (12)	0.0345 (5)	
H24	0.2597	0.0073	0.0803	0.041*	
C25	0.25608 (15)	0.2095 (2)	0.13743 (12)	0.0339 (5)	
H25	0.2886	0.1677	0.1860	0.041*	
C26	0.22641 (15)	0.3615 (2)	0.12975 (11)	0.0312 (5)	
H26	0.2376	0.4236	0.1732	0.037*	
C27	0.17899 (14)	0.4293 (2)	0.05797 (11)	0.0260 (4)	

### Atomic displacement parameters (Å^2^)

**Table:**

	*U*^11^	*U*^22^	*U*^33^	*U*^12^	*U*^13^	*U*^23^
N1	0.0431 (11)	0.0312 (10)	0.0275 (10)	−0.0035 (8)	0.0066 (8)	−0.0032 (8)
N2	0.0409 (10)	0.0305 (10)	0.0359 (10)	0.0027 (8)	0.0158 (9)	0.0012 (8)
N3	0.0388 (10)	0.0277 (9)	0.0353 (10)	0.0021 (8)	0.0148 (9)	0.0029 (8)
O1	0.0400 (10)	0.0717 (12)	0.0571 (11)	−0.0170 (9)	0.0088 (8)	−0.0273 (9)
O2	0.0602 (11)	0.0453 (9)	0.0393 (9)	0.0034 (8)	0.0155 (8)	−0.0165 (8)
O3	0.0253 (7)	0.0304 (8)	0.0328 (8)	−0.0026 (6)	0.0060 (6)	−0.0102 (6)
O4	0.0307 (8)	0.0404 (9)	0.0486 (9)	0.0042 (7)	0.0062 (7)	−0.0176 (7)
C1	0.0340 (11)	0.0229 (10)	0.0210 (10)	−0.0012 (8)	0.0040 (9)	0.0008 (8)
C2	0.0352 (12)	0.0302 (11)	0.0306 (11)	0.0052 (9)	0.0102 (9)	−0.0003 (9)
C3	0.0295 (11)	0.0335 (11)	0.0348 (12)	−0.0004 (9)	0.0074 (9)	0.0016 (9)
C4	0.0348 (12)	0.0224 (10)	0.0241 (10)	−0.0027 (9)	0.0023 (9)	0.0017 (8)
C5	0.0401 (12)	0.0276 (11)	0.0263 (11)	0.0024 (9)	0.0135 (9)	−0.0016 (9)
C6	0.0308 (11)	0.0299 (11)	0.0309 (11)	−0.0023 (9)	0.0106 (9)	0.0009 (9)
C7	0.0351 (12)	0.0226 (10)	0.0253 (11)	−0.0047 (9)	0.0013 (9)	0.0003 (8)
C8	0.0467 (13)	0.0280 (11)	0.0246 (11)	0.0042 (9)	0.0137 (10)	0.0006 (9)
C9	0.0327 (12)	0.0317 (11)	0.0296 (11)	−0.0027 (9)	0.0094 (9)	−0.0003 (9)
C10	0.0343 (11)	0.0202 (10)	0.0236 (10)	−0.0005 (8)	0.0045 (9)	−0.0015 (8)
C11	0.0316 (11)	0.0270 (10)	0.0282 (11)	−0.0010 (9)	0.0071 (9)	−0.0018 (9)
C12	0.0314 (11)	0.0301 (11)	0.0303 (11)	−0.0035 (9)	0.0056 (9)	−0.0022 (9)
C13	0.0271 (11)	0.0222 (10)	0.0337 (11)	−0.0021 (8)	0.0083 (9)	−0.0007 (9)
C14	0.0212 (10)	0.0246 (10)	0.0304 (11)	−0.0045 (8)	0.0069 (8)	−0.0032 (8)
C15	0.0202 (10)	0.0244 (10)	0.0343 (11)	−0.0031 (8)	0.0083 (9)	−0.0007 (9)
C16	0.0266 (11)	0.0249 (10)	0.0408 (13)	−0.0026 (8)	0.0091 (9)	−0.0020 (9)
C17	0.0350 (12)	0.0281 (11)	0.0482 (14)	0.0031 (9)	0.0092 (11)	0.0068 (10)
C18	0.0461 (14)	0.0416 (13)	0.0358 (13)	0.0042 (11)	0.0076 (11)	0.0094 (10)
C19	0.0422 (13)	0.0392 (13)	0.0316 (12)	0.0006 (10)	0.0105 (10)	−0.0004 (10)
C20	0.0256 (10)	0.0289 (11)	0.0318 (11)	−0.0015 (8)	0.0097 (9)	−0.0010 (9)
C21	0.0295 (11)	0.0299 (11)	0.0301 (11)	−0.0036 (9)	0.0108 (9)	−0.0062 (9)
C22	0.0213 (10)	0.0237 (10)	0.0319 (11)	−0.0034 (8)	0.0091 (9)	−0.0039 (8)
C23	0.0319 (11)	0.0251 (10)	0.0378 (12)	−0.0008 (9)	0.0142 (10)	−0.0050 (9)
C24	0.0336 (12)	0.0227 (10)	0.0466 (14)	−0.0001 (9)	0.0123 (10)	0.0022 (10)
C25	0.0313 (11)	0.0308 (11)	0.0353 (12)	−0.0014 (9)	0.0053 (9)	0.0056 (9)
C26	0.0319 (11)	0.0280 (11)	0.0314 (12)	−0.0040 (9)	0.0071 (9)	−0.0009 (9)
C27	0.0209 (10)	0.0248 (10)	0.0316 (11)	−0.0044 (8)	0.0078 (8)	−0.0019 (8)

### Geometric parameters (Å, °)

**Table:**

N1—O1	1.223 (2)	C12—H12	0.9500
N1—O2	1.223 (2)	C13—C14	1.494 (3)
N1—C1	1.467 (2)	C14—C15	1.409 (3)
N2—N3	1.231 (2)	C14—C27	1.416 (3)
N2—C4	1.445 (2)	C15—C16	1.434 (3)
N3—C7	1.443 (2)	C15—C20	1.437 (3)
O3—C13	1.365 (2)	C16—C17	1.354 (3)
O3—C10	1.402 (2)	C16—H16	0.9500
O4—C13	1.196 (2)	C17—C18	1.413 (3)
C1—C2	1.373 (3)	C17—H17	0.9500
C1—C6	1.385 (3)	C18—C19	1.349 (3)
C2—C3	1.377 (3)	C18—H18	0.9500
C2—H2	0.9500	C19—C20	1.420 (3)
C3—C4	1.381 (3)	C19—H19	0.9500
C3—H3	0.9500	C20—C21	1.389 (3)
C4—C5	1.391 (3)	C21—C22	1.389 (3)
C5—C6	1.378 (3)	C21—H21	0.9500
C5—H5	0.9500	C22—C23	1.426 (3)
C6—H6	0.9500	C22—C27	1.429 (3)
C7—C12	1.379 (3)	C23—C24	1.352 (3)
C7—C8	1.387 (3)	C23—H23	0.9500
C8—C9	1.393 (3)	C24—C25	1.411 (3)
C8—H8	0.9500	C24—H24	0.9500
C9—C10	1.384 (3)	C25—C26	1.361 (3)
C9—H9	0.9500	C25—H25	0.9500
C10—C11	1.385 (3)	C26—C27	1.423 (3)
C11—C12	1.374 (3)	C26—H26	0.9500
C11—H11	0.9500		
			
O1—N1—O2	123.42 (18)	O3—C13—C14	109.61 (15)
O1—N1—C1	118.41 (17)	C15—C14—C27	121.42 (17)
O2—N1—C1	118.17 (18)	C15—C14—C13	118.08 (16)
N3—N2—C4	111.35 (17)	C27—C14—C13	120.48 (17)
N2—N3—C7	113.66 (17)	C14—C15—C16	124.09 (18)
C13—O3—C10	123.10 (14)	C14—C15—C20	118.80 (17)
C2—C1—C6	122.62 (18)	C16—C15—C20	117.09 (17)
C2—C1—N1	118.71 (17)	C17—C16—C15	121.37 (19)
C6—C1—N1	118.67 (17)	C17—C16—H16	119.3
C1—C2—C3	118.39 (19)	C15—C16—H16	119.3
C1—C2—H2	120.8	C16—C17—C18	120.84 (19)
C3—C2—H2	120.8	C16—C17—H17	119.6
C2—C3—C4	120.25 (19)	C18—C17—H17	119.6
C2—C3—H3	119.9	C19—C18—C17	120.1 (2)
C4—C3—H3	119.9	C19—C18—H18	119.9
C3—C4—C5	120.56 (18)	C17—C18—H18	119.9
C3—C4—N2	114.30 (17)	C18—C19—C20	121.3 (2)
C5—C4—N2	125.14 (18)	C18—C19—H19	119.3
C6—C5—C4	119.65 (18)	C20—C19—H19	119.3
C6—C5—H5	120.2	C21—C20—C19	121.55 (18)
C4—C5—H5	120.2	C21—C20—C15	119.19 (18)
C5—C6—C1	118.43 (18)	C19—C20—C15	119.26 (18)
C5—C6—H6	120.8	C22—C21—C20	122.30 (18)
C1—C6—H6	120.8	C22—C21—H21	118.8
C12—C7—C8	120.27 (18)	C20—C21—H21	118.8
C12—C7—N3	114.06 (17)	C21—C22—C23	121.17 (17)
C8—C7—N3	125.67 (18)	C21—C22—C27	119.68 (17)
C7—C8—C9	120.27 (18)	C23—C22—C27	119.15 (18)
C7—C8—H8	119.9	C24—C23—C22	121.19 (19)
C9—C8—H8	119.9	C24—C23—H23	119.4
C10—C9—C8	118.32 (19)	C22—C23—H23	119.4
C10—C9—H9	120.8	C23—C24—C25	119.90 (19)
C8—C9—H9	120.8	C23—C24—H24	120.0
C9—C10—C11	121.49 (18)	C25—C24—H24	120.0
C9—C10—O3	125.30 (17)	C26—C25—C24	120.85 (19)
C11—C10—O3	113.16 (16)	C26—C25—H25	119.6
C12—C11—C10	119.47 (18)	C24—C25—H25	119.6
C12—C11—H11	120.3	C25—C26—C27	121.29 (19)
C10—C11—H11	120.3	C25—C26—H26	119.4
C11—C12—C7	120.18 (19)	C27—C26—H26	119.4
C11—C12—H12	119.9	C14—C27—C26	123.82 (17)
C7—C12—H12	119.9	C14—C27—C22	118.54 (17)
O4—C13—O3	123.49 (17)	C26—C27—C22	117.60 (17)
O4—C13—C14	126.81 (18)		

### Hydrogen-bond geometry (Å, °)

**Table:**

*D*—H···*A*	*D*—H	H···*A*	*D*···*A*	*D*—H···*A*
C26—H26···O2^i^	0.95	2.54	3.273 (3)	134
C17—H17···O4^ii^	0.95	2.57	3.509 (3)	169

Symmetry codes: (i) −*x*+1, −*y*+2, −*z*+1; (ii) −*x*, −*y*+2, −*z*.
